# Sex-specific differences in the association between triglyceride glucose index and carotid plaque in a cardiovascular high-risk population: a cross-sectional study based on a Chinese community-dwelling population

**DOI:** 10.3389/fcvm.2024.1473171

**Published:** 2024-10-15

**Authors:** Weiguo Lin, Mengjie Xu, Jinbiao Zheng, Ruixue Sun, Shaorong Yan, Xiaoshu Chen, Yuzhan Lin

**Affiliations:** ^1^ Department of Urology, The Third Affiliated Hospital of Wenzhou Medical University, Ruian, Zhejiang, China; ^2^ Department of Laboratory Medicine, Wenzhou People’s Hospital, The Third Affiliated Hospital of Shanghai University, The Wenzhou Third Clinical College of Wenzhou Medical University, Wenzhou, Zhejiang, China; ^3^ Department of Clinical Laboratory, The Third Affiliated Hospital of Wenzhou Medical University, Ruian, Zhejiang, China; ^4^ Department of Cardiology, Wenzhou People’s Hospital, The Third Affiliated Hospital of Shanghai University, The Wenzhou Third Clinical College of Wenzhou Medical University, Wenzhou, Zhejiang, China

**Keywords:** triglyceride glucose index, carotid plaque, sex differences, cardiovascular risk, generalized additive model

## Abstract

**Background:**

To date, numerous studies have investigated the relationship between the triglyceride glucose (TyG) index and carotid plaques, but the impact of gender on this relationship has not been explored. Therefore, this study aims to investigate gender-specific differences in the relationship between the TyG index and carotid plaques in a high cardiovascular risk population in China.

**Methods:**

This cross-sectional study's data were derived from a longitudinal pilot study involving 1,085 high-risk cardiovascular subjects. A multivariable logistic regression model was used to analyze the relationship between the TyG index and carotid plaques. A generalized additive model combined with a stratified regression model was employed to assess the nonlinear relationship between the TyG index and carotid plaques across different genders. In the nonlinear relation, the inflection point was calculated by a two-piecewise linear regression model.

**Results:**

After adjusting for confounders such as age, sex, BMI, SBP, DBP, AST/ALT, TC, LDL-c, HDL-c, creatinine, smoking, and antilipemic medication, the generalized additive model results revealed a nonlinear relationship between the TyG index and carotid plaque formation, with significant differences across genders. In males, the relationship between the TyG index and carotid plaques was S-shaped. The two-piecewise linear regression model identified two inflection points: TyG = 8.39 (*P* = 0.017) and TyG = 10.2 (*P* = 0.009).

**Conclusion:**

The relationship between the TyG index and the formation of carotid plaques is nonlinear, and there are significant differences in the correlation between males and females.

## Introduction

Reports indicate a rising global prevalence of carotid plaques, reaching 21.1% in 2020, underscoring the growing concern over this condition (1). The advanced stage of carotid atherosclerosis involves the formation of arterial plaques. If these plaques detach, they may obstruct blood vessels, potentially leading to ischemic stroke (2). Moreover, the progression of atherosclerosis is insidious and slow, with no clinical symptoms in the early stages, but it can present suddenly with cerebral ischemia-related conditions such as hemiplegia and aphasia during acute episodes (3). Carotid atherosclerosis is now widely recognized as a marker for systemic atherosclerosis and a predictive indicator for clinical cardiovascular events (4). Despite the high incidence and poor prognosis of carotid plaques, the associated risk factors have not been thoroughly investigated. Therefore, identifying and managing these risk factors is crucial for proactive prevention at the subclinical stage.

Among the various risk factors, studies have shown a positive correlation between the Triglyceride Glucose (TyG) index and the incidence of cardiovascular and cerebrovascular diseases (5–7). The TyG index, calculated from serum triglyceride and fasting blood glucose (FBG) levels, is widely recommended as an alternative indicator of insulin resistance due to its stability and reliability (8, 9). In the field of type 2 diabetes, the link between the formation of carotid plaques and insulin resistance has been well established (10). Based on the association between the TyG index and insulin resistance, as well as the role of insulin resistance in the formation of carotid plaques, researchers have hypothesized a potential correlation between the TyG index and carotid plaques.

Numerous studies have explored the relationship between the TyG index and carotid plaques, including large cross-sectional studies analyzing their association (11, 12), longitudinal studies examining their causal relationship (13–16), and research indicating a nonlinear correlation between the two (17). However, the relationship between the TyG index and carotid plaques across different genders remains unclear (11, 18). As previously reported (19), glucose and lipid metabolism differ between genders. Therefore, we hypothesize that the relationship between the TyG index and carotid plaques differs between genders. The aim of this study is to explore the gender-specific differences in the relationship between the TyG index and carotid plaques in a high cardiovascular risk population in China.

## Methods

### Study population and design

The study population for this cross-sectional study was identified from high-risk cardiovascular disease subjects in the pilot screening survey of the longitudinal study (China PEACE Million Persons Project) initiated by the National Center for Cardiovascular Disease in Zhejiang Province, Wenzhou City, from 2019 to 2020 (20). This project was jointly approved by the Central Ethics Committee of the National Center for Cardiovascular Diseases and the Ethics Committee of Wenzhou People's Hospital. Since its inception in 2014, the China PEACE Million Persons Project has been continuously carried out nationwide, increasing to 383 project sites and screening over a million people by 2023. The main screening targets of the study were permanent residents of the project sites, with the inclusion criteria being aged between 35 and 75 years old, and having lived in the selected area for at least six months in the past 12 months with formal household registration (a formal record for identifying residents of the area). All participants provided written informed consent. Data were missing for less than 5% of participants, and audit results indicated high overall data quality.

### Screening of high-risk subjects for cardiovascular disease

In the preliminary screening phase, experienced project team members, trained in cardiovascular health, identified participants’ cardiovascular risk factors through initial assessments, including inquiries about cardiovascular health history, physical examinations, and rapid blood glucose and lipid level tests. They assessed the likelihood of participants developing cardiovascular diseases to determine if they were part of the high-risk group. Participants were considered high-risk individuals if they met any of the following criteria:

(1) Past medical history (meeting any of the following conditions is sufficient):
  (a) History of myocardial infarction;
  (b) Undergone percutaneous coronary intervention;
  (c) Undergone coronary artery bypass graft surgery;
  (d) History of stroke, including ischemic or hemorrhagic types.
(2) Blood pressure and blood lipid level standards (meeting any of the following conditions is sufficient):
  (a) Systolic blood pressure reaching or exceeding 160 mmHg or diastolic blood pressure reaching or exceeding 100 mmHg;
  (b) LDL cholesterol levels reaching or exceeding 160 mg/dl (4.14 mmol/L);
  (c) HDL cholesterol levels below 30 mg/dl (0.78 mmol/L).
(3) Cardiovascular disease risk assessment:

According to the risk assessment tool in the “Cardiovascular Risk Assessment and Management Guidelines” published by the World Health Organization in 2008 (21), all participants were assessed for risk. Those with a risk of developing cardiovascular diseases within the next ten years of 20% or more were considered high-risk individuals.

The assessment considered the following factors:

(a) Age;
(b) Gender;
(c) Systolic blood pressure (average of two measurements, unit: millimeters of mercury);
(d) Smoking status (current smokers or those who have quit smoking within the past year are both considered smokers);
(e) Diabetes status (previous diagnosis, use of hypoglycemic medication, or using insulin);
(f) Total blood cholesterol levels (in mmol/L).

### Data collection and definitions

During general examinations, general characteristics of participants were recorded, including age, gender, body mass index (BMI), systolic blood pressure (SBP), and diastolic blood pressure (DBP). In blood tests, blood glucose levels (BG), triglycerides (TG), glycated hemoglobin (HbA 1c), low-density lipoprotein cholesterol (LDL-C), and high-density lipoprotein cholesterol (HDL-C) were included, using fasting blood samples. Participants’ long-term medication history (antihypertensive drugs, lipid-lowering drugs, hypoglycemic drugs) and lifestyle (including smoking and drinking habits) were obtained through standardized self-administered questionnaires. Hypertension is defined as self-reported hypertension, use of antihypertensive drugs, SBP ≥ 140 mmHg, DBP ≥ 90 mmHg (or both) (22, 23). Diabetes is defined as the use of hypoglycemic drugs, glycated hemoglobin levels ≥6.5%, and fasting blood glucose levels ≥7 mmol/L (24). According to the definition by the National Institute on Alcohol Abuse and Alcoholism, drinking habits are divided into never drinking, light drinking (1–2 cups/day), moderate drinking (3–4 cups/day), or heavy drinking (>5 cups/day) (25). Smoking status is divided into non-smoker, former smoker, and current smoker.

### Tyg calculation formula

According to previous studies (26), the formula for calculating the TyG index in this study is as follows:

$$\mathrm{TyG}=\ln\;\left(\frac{\mathrm{TG}\;\left(\mathrm{mg}/\mathrm{dL}\right)\times \;\mathrm{FBG}\;\left(\mathrm{mg}/\mathrm{dL}\right)\;}{2}\right)$$

### Carotid plaque measurement

Carotid ultrasound examinations were conducted by trained and experienced sonographers who were blinded to the baseline characteristics and laboratory test results of the subjects. Equipment settings included a high-end color Doppler ultrasound device with a linear array transducer at a frequency of 5–12 MHz. Observation site: the actual observation of the entire carotid artery plaque occurrence, including the internal carotid artery, external carotid artery, carotid bifurcation, and the main trunk of the common carotid artery. Examination process: first take the transverse section, then take the longitudinal section, starting from the proximal end of the common carotid artery, from near to far, gradually sweeping towards the distal end, observing the intima-media display of the common carotid artery, bifurcation, internal and external carotid arteries. First, check the left side, then the right side. Intima-media thickness (IMT) definition: refers to the vertical distance between the upper edge of the intima and the lower edge of the media of the carotid artery (27). Carotid plaque is defined as local thickening of the intima-media ≥1.5 millimeters or thickening >50% of the surrounding IMT value (28).

### Statistical analysis

Participants were categorized into four groups based on the quartiles of the TyG index, and their characteristics were described. Categorical variables were represented by numbers (N) and percentages (%), and group comparisons were assessed using the chi-square test. Continuous variables were described using means and standard deviations if they were normally distributed; otherwise, medians and interquartile ranges were used. Group comparisons for normally distributed continuous variables were made using one-way analysis of variance, while the Mann–Whitney *U* test was used for non-normally distributed continuous variables.

Single-factor and multiple-factor logistic regression models were employed to analyze the relationship between the TyG index and carotid plaque. Model Ⅰ was adjusted for age, sex, BMI, smoking, drinking, and waistline. Model Ⅱ further adjusted for additional physiological and biochemical indicators, including blood pressure, liver function indicators (AST/ALT), blood lipid levels (TC, LDL-C, HDL-C), and renal function indicators (creatinine), antilipidemic medication use, as well as socioeconomic factors such as education level and occupation, to more comprehensively assess the association between the TyG index and carotid plaque and control for potential confounding variables. The selection of confounding factors was based on three principles: first, variables were chosen according to their clinical significance; second, relevant studies were reviewed for reference (12, 29, 30); and third, covariates were adjusted if their inclusion changed the odds ratios by less than 10% (31). These three criteria were used to determine the necessary adjustments for confounding factors.

A generalized additive model was utilized to evaluate the nonlinear relationship between the TyG index and carotid plaques. In cases where a nonlinear association was detected, a two-piece linear regression model was applied to calculate the threshold effect of the TyG index on carotid plaques (32). When a clear relationship between the TyG index and carotid plaques was evident on a smoothed curve, recursive methods were automatically employed by the software to determine inflection points after adjusting for potential confounders. A likelihood ratio test was used to examine the interaction effect of gender on the relationship between the TyG index and carotid plaques. The statistical analyses of this study were performed via R, version 4.2.0 (R Foundation) and EmpowerStats (http://www.empowerstats.com, X&Y Solutions, Inc., Boston, MA). The level of statistical significance was set at *p* < 0.05.

## Results

### Baseline characteristics based on TyG index tertiles

**Figure 1:**
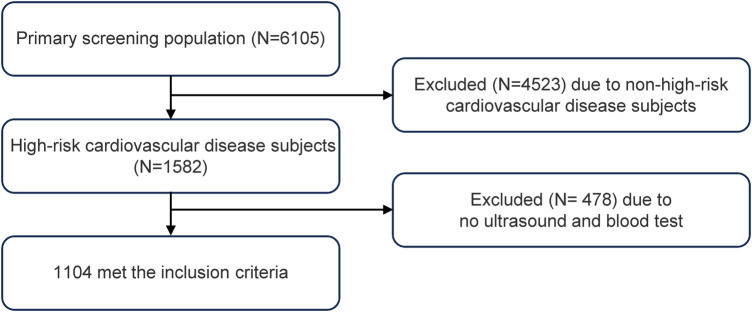
Flowchart of the subject selection.

This cross-sectional study evaluated a total of 1,085 participants (Figure 1). The quartiles of the TyG index were used for classification (Table 1). The TyG index increased significantly with each ascending quartile, and concurrent significant changes were observed in waist circumference, BMI, systolic and diastolic blood pressure, ALT and AST levels, blood glucose, HDL-c, LDL-c, TC, and TG. The distribution of gender indicated a higher proportion of male participants with higher TyG index values. The prevalence of smoking and drinking habits, as well as hypertension and diabetes, also significantly changed with an increase in the TyG index. In addition, the use of antihypertensive and antidiabetic medications became more common with the increase in the TyG index. Associations between urinary protein levels, occupation type, and education level with the TyG index were not significant or only had weak statistical relevance.

**Table 1:** Baseline characteristics of participants (*N* = 1,085).

Characteristic	TyG index (quartiles)				
	Q1	Q2	Q3	Q4	*P* value
Number	271	271	271	272	
TyG index	8.18 ± 0.26	8.70 ± 0.11	9.08 ± 0.12	9.75 ± 0.43	<0.001
Carotid plaque (*n*, %)					0.155
No	191 (70.48)	171 (63.10)	172 (63.47)	169 (62.13)	
Yes	80 (29.52)	100 (36.90)	99 (36.53)	103 (37.87)	
Age, years	61.12 ± 9.60	62.08 ± 8.73	62.83 ± 8.78	61.27 ± 8.11	0.088
Sex (*n*, %)					<0.001
Female	185 (68.27)	163 (60.15)	164 (60.52)	116 (42.65)	
Male	86 (31.73)	108 (39.85)	107 (39.48)	156 (57.35)	
Waistline, cm	81.13 ± 8.11	85.36 ± 9.01	86.92 ± 8.38	88.34 ± 8.17	<0.001
BMI, kg/m^2^	23.24 ± 2.86	24.82 ± 3.24	25.35 ± 2.91	25.51 ± 2.98	<0.001
SBP, mmHg	153.30 ± 24.16	157.35 ± 21.00	158.73 ± 21.36	162.10 ± 17.77	<0.001
DBP, mmHg	83.51 ± 12.85	86.69 ± 11.79	87.30 ± 13.09	90.42 ± 11.86	<0.001
CHOPE/per week, hours	7.17 ± 5.71	7.70 ± 5.21	8.49 ± 5.06	7.89 ± 6.30	0.393
Education (*n*, %)					0.433
Below high school	251 (92.62)	256 (94.46)	258 (95.20)	251 (92.28)	
Above high school	20 (7.38)	15 (5.54)	13 (4.80)	21 (7.72)	
Occupation (*n*, %)					0.545
Nonagricultural laborer	249 (91.88)	239 (88.19)	244 (90.04)	243 (89.34)	
Agricultural laborer	22 (8.12)	32 (11.81)	27 (9.96)	29 (10.66)	
Smoking (*n*, %)					<0.001
Never	230 (84.87)	212 (78.23)	211 (77.86)	182 (66.91)	
Now	23 (8.49)	39 (14.39)	34 (12.55)	52 (19.12)	
Ever	18 (6.64)	20 (7.38)	26 (9.59)	38 (13.97)	
Alcohol (*n*, %)					0.273
Never	176 (64.94)	173 (63.84)	166 (61.25)	150 (55.15)	
Light drinking	79 (29.15)	81 (29.89)	80 (29.52)	96 (35.29)	
Moderate drinking	12 (4.43)	11 (4.06)	13 (4.80)	17 (6.25)	
Excessive drinking	4 (1.48)	6 (2.21)	12 (4.43)	9 (3.31)	
Hypertension (*n*, %)					<0.001
No	57 (21.03)	33 (12.18)	40 (14.76)	18 (6.62)	
Yes	214 (78.97)	238 (87.82)	231 (85.24)	254 (93.38)	
Diabetes (*n*, %)					<0.001
No	241 (88.93)	195 (71.96)	180 (66.42)	135 (49.63)	
Yes	30 (11.07)	76 (28.04)	91 (33.58)	137 (50.37)	
Antihypertension medication (*n*, %)					<0.001
No	107 (50.00)	93 (39.08)	73 (31.60)	87 (34.25)	
Yes	107 (50.00)	145 (60.92)	158 (68.40)	167 (65.75)	
Antidiabetic medication (*n*, %)					0.745
No	8 (26.67)	27 (35.53)	34 (37.36)	46 (33.58)	
Yes	22 (73.33)	49 (64.47)	57 (62.64)	91 (66.42)	
Antilipemic medication (*n*, %)					0.096
No	241 (88.93)	224 (82.66)	222 (81.92)	226 (83.09)	
Yes	30 (11.07)	47 (17.34)	49 (18.08)	46 (16.91)	
History of myocardial infarction (*n*, %)					0.572
No	271 (100.00)	270 (99.63)	271 (100.00)	271 (99.63)	
Yes	0 (0.00)	1 (0.37)	0 (0.00)	1 (0.37)	
History of PCI (*n*, %)					0.070
No	266 (98.15)	269 (99.26)	270 (99.63)	272 (100.00)	
Yes	5 (1.85)	2 (0.74)	1 (0.37)	0 (0.00)	
History of CABG (*n*, %)					0.281
No	270 (99.63)	268 (98.89)	270 (99.63)	272 (100.00)	
Yes	1 (0.37)	3 (1.11)	1 (0.37)	0 (0.00)	
History of stroke (*n*, %)					0.466
No	268 (98.89)	266 (98.15)	265 (97.79)	270 (99.26)	
Yes	3 (1.11)	5 (1.85)	6 (2.21)	2 (0.74)	
History of coronary heart disease (*n*, %)					0.131
No	265 (97.79)	265 (97.79)	269 (99.26)	271 (99.63)	
Yes	6 (2.21)	6 (2.21)	2 (0.74)	1 (0.37)	
History of stenocardia (*n*, %)					0.953
No	269 (99.26)	269 (99.26)	268 (98.89)	270 (99.26)	
Yes	2 (0.74)	2 (0.74)	3 (1.11)	2 (0.74)	
ALT, U/L	20.05 ± 10.96	24.18 ± 13.11	27.30 ± 17.57	31.47 ± 26.73	<0.001
AST, U/L	24.25 ± 8.54	25.51 ± 9.74	26.27 ± 12.37	30.43 ± 41.89	0.011
AST/ALT	1.33 ± 0.44	1.18 ± 0.44	1.07 ± 0.33	1.02 ± 0.35	<0.001
Creatinine, mmol/L	70.42 ± 20.09	75.26 ± 57.68	72.80 ± 18.44	77.00 ± 21.12	0.115
BG, mmol/L	5.63 ± 0.68	5.96 ± 0.95	6.42 ± 1.42	7.53 ± 2.67	<0.001
HDL-c, mmol/L	1.48 ± 0.31	1.32 ± 0.27	1.28 ± 0.31	1.15 ± 0.26	<0.001
LDL-c, mmol/L	2.94 ± 0.82	3.06 ± 0.95	3.10 ± 0.90	2.81 ± 0.91	<0.001
TC, mmol/L	5.01 ± 1.00	5.17 ± 1.17	5.36 ± 1.12	5.50 ± 1.24	<0.001
TG, mmol/L	0.82 ± 0.18	1.30 ± 0.22	1.79 ± 0.37	3.39 ± 2.20	<0.001
Urinary protein (*n*, %)					0.012
−	262 (97.04)	256 (94.46)	249 (92.22)	239 (87.87)	
±	3 (1.11)	3 (1.11)	4 (1.48)	5 (1.84)	
1+	2 (0.74)	9 (3.32)	7 (2.59)	10 (3.68)	
2+	3 (1.11)	3 (1.11)	9 (3.33)	17 (6.25)	
3+	0 (0.00)	0 (0.00)	1 (0.37)	1 (0.37)	
Cardiovascular high-risk criteria as follow (*n*, %)					
Criteria 1					0.200
No	262 (96.68)	260 (95.94)	263 (97.05)	269 (98.90)	
Yes	9 (3.32)	11 (4.06)	8 (2.95)	3 (1.10)	
Criteria 2.1					0.009
No	100 (36.90)	84 (31.00)	80 (29.52)	64 (23.53)	
Yes	171 (63.10)	187 (69.00)	191 (70.48)	208 (76.47)	
Criteria 2.2					0.114
No	169 (62.36)	178 (65.68)	181 (66.79)	196 (72.06)	
Yes	102 (37.64)	93 (34.32)	90 (33.21)	76 (27.94)	
Criteria 3					<0.001
No	237 (87.45)	226 (83.39)	212 (78.23)	195 (71.69)	
Yes	34 (12.55)	45 (16.61)	59 (21.77)	77 (28.31)	

Values are given as mean ± SD, medians with IQR or number (%).

Criteria 1: past history of diseases; Criteria 2.1: meet blood pressure level standards; Criteria 2.2: meet blood lipid level standards; Criteria 3: cardiovascular disease risk within the next ten years of 20% or more.

TyG, triglyceride glucose; BMI, body mass index; SBP, systolic blood pressure; DBP, diastole blood pressure; CHOPE, cumulative hours of physical exercise; PCI, percutaneous coronary intervention; CABG, coronary artery bypass grafting; ALT, alanine aminotransferase; AST, aspartate aminotransferase; BG, blood glucose; HDL-c, high-density lipoprotein cholesterol; LDL-c, low-density lipoprotein cholesterol; TC, total cholesterol; TG, triglyceride.

### Relationship between TyG index and carotid plaque under different models

Firstly, potential risk factors or protective factors were included in the univariate logistic regression analysis. In the results, the TyG index, gender, age, waist circumference, systolic blood pressure, diastolic blood pressure, creatinine, and blood glucose levels were significantly associated with carotid plaque. When included as a continuous variable, an increase of one unit in the TyG index increased the risk of carotid plaque formation by 30% (OR:1.30, 95% CI: 1.07–1.58, *p* = 0.009) (Table 2). Subsequently, to assess the degree of association between the TyG index and carotid plaque, a multivariate logistic regression analysis was conducted. Table 3 demonstrated the relationship between the TyG index and carotid plaque using a multivariate logistic regression model. In Model I, the association between the TyG index and carotid plaque was slightly weakened but remained significant (OR 1.30, 95% CI 1.03–1.64, *P* = 0.028). However, in Model II, which further adjusted for smoking, alcohol, education, occupation, SBP, DBP, AST/ALT, creatinine, TC, HDL-c, LDL-c, antilipemic medication, antihypertension medication, diabetes, urinary protein and history of myocardial infarction, the association between the TyG index and carotid plaque was no longer significant (OR = 1.46, 95% CI 0.98–2.18, *P* = 0.066).

**Table 2:** Univariate analysis for carotid plaque.

Covariate	Statistics	OR (95%CI) *P*-value
TyG	8.9 ± 0.6	1.30 (1.07, 1.58) 0.009
Sex		
Female	644 (58.33)	Ref.
Male	460 (41.67)	2.66 (2.06, 3.43) <0.001
Age	61.87 ± 8.83	1.11 (1.09, 1.12) <0.001
BMI	24.74 ± 3.12	0.98 (0.94, 1.02) 0.335
Waistline	85.46 ± 8.84	1.03 (1.01, 1.04) <0.001
SBP	157.96 ± 21.44	1.02 (1.01, 1.02) <0.001
DBP	86.95 ± 12.60	1.00 (0.99, 1.01) 0.573
ALT	25.75 ± 18.60	1.00 (1.00, 1.01) 0.530
AST	26.62 ± 22.89	1.01 (1.00, 1.01) 0.196
AST/ALT	1.15 ± 0.41	1.22 (0.90, 1.64) 0.199
Creatinine,	73.87 ± 33.64	1.01 (1.01, 1.02) <0.001
BG	6.38 ± 1.77	1.15 (1.07, 1.23) <0.001
HDL-c	1.31 ± 0.31	0.97 (0.65, 1.45) 0.888
LDL-c	2.98 ± 0.90	1.02 (0.88, 1.17) 0.825
TC	5.26 ± 1.15	1.02 (0.92, 1.14) 0.655
TG	1.83 ± 1.48	1.06 (0.98, 1.15) 0.151
Smoking		
Never	853 (77.26)	Ref.
Now	148 (13.41)	3.33 (2.33, 4.76) <0.001
Ever	103 (9.33)	2.09 (1.38, 3.17) <0.001
Alcohol		
Never	678 (61.41)	Ref.
Light drinking	339 (30.71)	1.34 (1.02, 1.75) 0.037
Moderate drinking	54 (4.89)	1.32 (0.74, 2.33) 0.345
Excessive drinking	33 (2.99)	0.90 (0.42, 1.92) 0.784
Occupation		
Nonagricultural laborer	993 (89.95)	Ref.
Agricultural laborer	111 (10.05)	1.55 (1.04, 2.30) 0.032
Education		
Below high school	1,034 (93.66)	Ref.
Above high school	70 (6.34)	0.41 (0.22, 0.75) 0.004
Hypertension		
No	151 (13.68)	Ref.
Yes	953 (86.32)	2.91 (1.87, 4.52) <0.001
Diabetes		
No	766 (69.38)	Ref.
Yes	338 (30.62)	1.74 (1.34, 2.26) <0.001
Antihypertension medication		
No	368 (38.61)	Ref.
Yes	585 (61.39)	1.88 (1.43, 2.49) <0.001
Antidiabetic medication		
No	118 (34.91)	Ref.
Yes	220 (65.09)	1.51 (0.95, 2.38) 0.079
Antilipemic medication		
No	931 (84.33)	Ref.
Yes	173 (15.67)	1.77 (1.27, 2.45) <0.001
Urinary protein		
−	1,006 (92.89)	Ref.
±	15 (1.39)	1.72 (0.62, 4.79) 0.298
1+	28 (2.59)	1.48 (0.69, 3.15) 0.316
2+	32 (2.95)	4.33 (2.03, 9.25) <0.001
3+	2 (0.18)	0.00 (0.00, Inf) 0.9,723

Values are given as mean ± SD, medians with IQR or number (%).

CI, confidence interval; OR, odds ratio.

Inf: The model failed because of the small sample size.

**Table 3:** Relationship between TyG and carotid plaque.

Exposure	Non-adjusted OR (95%CI) *P*-value	Model I OR (95%CI) *P*-value	Model II OR (95%CI) *P*-value
TyG	1.30 (1.07, 1.58) 0.009	1.30 (1.03, 1.64) 0.028	1.46 (0.98, 2.18) 0.066
TyG (quartile)			
Q1	Ref	Ref	Ref
Q2	1.40 (0.97, 2.00) 0.069	1.37 (0.91, 2.07) 0.136	1.36 (0.85, 2.16) 0.196
Q3	1.37 (0.96, 1.97) 0.083	1.34 (0.88, 2.03) 0.172	1.25 (0.76, 2.06) 0.383
Q4	1.46 (1.02, 2.08) 0.040	1.40 (0.92, 2.13) 0.116	1.28 (0.72, 2.28) 0.395
P for trend	0.057	0.165	0.521

Non-adjusted model adjusted for: None.

Model I adjusted for age, sex, BMI, smoking, alcohol and waistline.

Model II adjusted for age, sex, BMI, waistline, smoking, alcohol, education, occupation, SBP, DBP, AST/ALT, creatinine, TC, HDL-c, LDL-c, antilipemic medication, antihypertension medication, diabetes, urinary protein and history of myocardial infarction.

### Analysis of the nonlinear relationship between TyG index and carotid plaque in different gender

**Figure 2:**
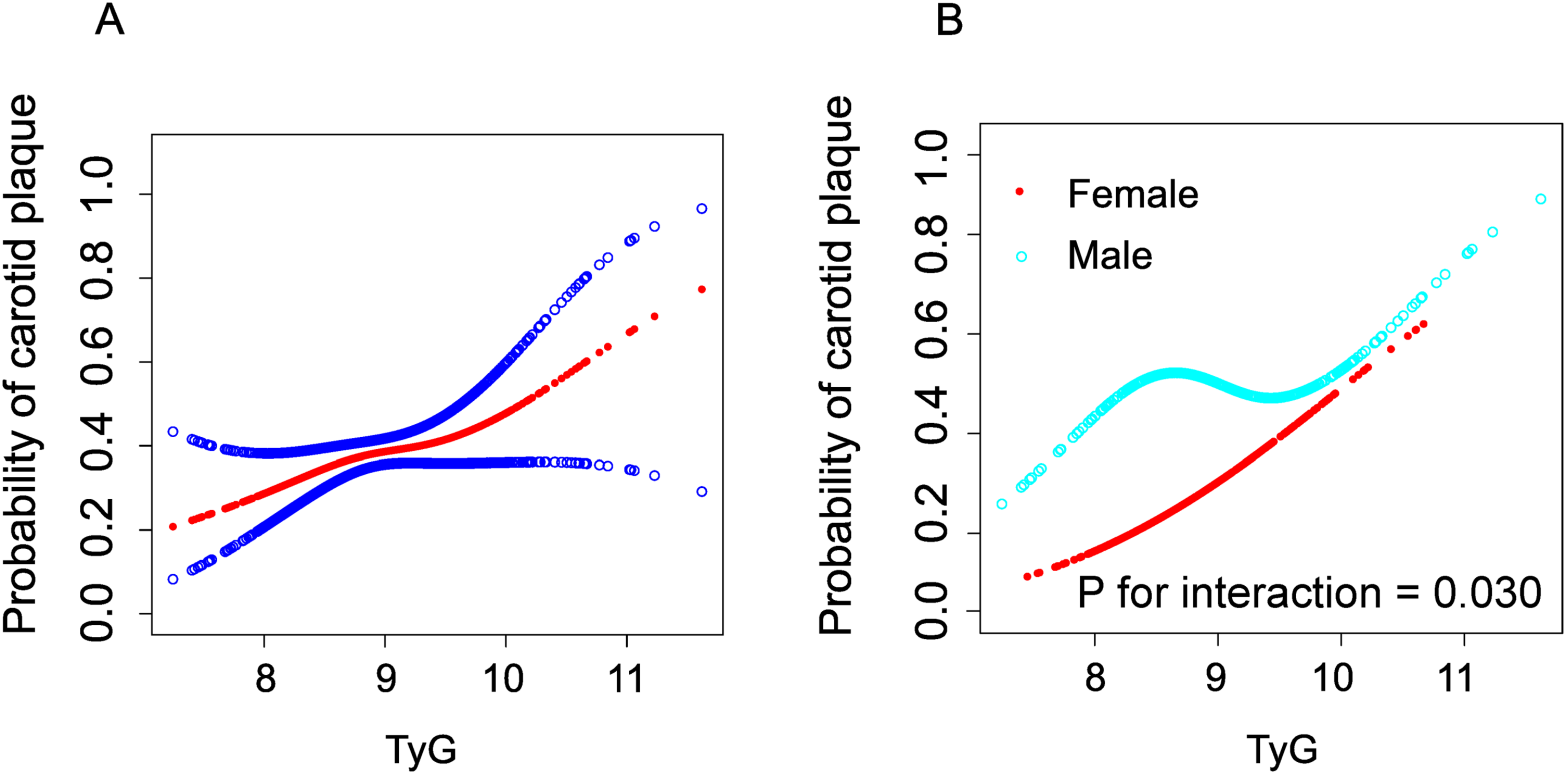
Construction of smooth curve describing the risk of carotid plaque against TyG Index using generalisedadditive model. The red line in **(A)** represents a smooth curve fit between variables. The blue line indicates a 95% Cl. In a **(B)**, the red line represents the female and the blue line represents the male. Adjustment variables include age, sex, BMI, waistline, smoking, alcohol, education, occupation, SBP, DBP, AST/ALT, creatinine, TC, HDL-c, LDL-c, antilipemic medication, antihypertension medication, diabetes, urinary protein and history of myocardial infarction.

We employed generalized additive models (GAM) and smooth curve fitting to further investigate the potential nonlinear relationship between the TyG index and carotid plaques in the overall population and across different gender groups (adjusted for age, sex, BMI, waistline, smoking, alcohol, education, occupation, SBP, DBP, AST/ALT, creatinine, TC, HDL-c, LDL-c, antilipemic medication, antihypertension medication, diabetes, urinary protein and history of myocardial infarction) (Figure 2). Figure 2A illustrates that as the TyG index increases, the probability of forming carotid plaques also increases and exhibits a nonlinear correlation. Interestingly, as shown in Figure 2B, unlike in females, the relationship between the TyG index and carotid plaques in males follows an S-shaped curve. The interaction effect of gender was statistically significant (*P* = 0.030). By using a recursive algorithm, we further identified two different inflection points (8.42, *P* = 0.008 and 9.23, *P* = 0.079) (Table 4). When the TyG index is below 8.42, each unit increase is significantly associated with an increased risk of carotid plaque (OR = 5.63, 95% CI 1.16–27.26, *P* = 0.032); however, when the TyG index is above 8.42, the risk increase is not significant (*P* = 0.055). Another inflection point occurs at 9.23, where the risk ratio for values below this threshold is 1.06, which is not significant (*P* = 0.805), whereas for values above 9.23, the risk significantly escalates to 5.68 (95% CI:1.58–20.47, *P* = 0.008).

**Table 4:** Threshold effect analysis of TyG and carotid plaque in male.

Inflection point	OR (95%CI)	*P*-value
K1		
Inflection point	8.42	
TyG Index <K1	5.63 (1.16, 27.26)	0.032
TyG Index >K1	0.38 (0.14, 1.02)	0.055
P for log likelihood ratio test		0.008
K2		
Inflection point	9.23	
TyG index <K2	1.06 (0.65, 1.74)	0.805
TyG index >K2	5.68 (1.58, 20.47)	0.008
P for log likelihood ratio test		0.079

Age, BMI, waistline, smoking, alcohol, education, occupation, SBP, DBP, AST/ALT, creatinine, TC, HDL-c, LDL-c, antilipemic medication, antihypertension medication, diabetes, urinary protein and history of myocardial infarction.

### Interaction test results across different subgroups

The effect modification of gender on the relationship between TyG and carotid plaques across different subgroups is presented in Supplementary Figure S1. The results indicate that gender differences persist across various subgroups. In the hypertensive population, the interaction between gender and TyG is significant (P for interaction = 0.029).

## Discussion

This cross-sectional study found a nonlinear association between the TyG index and the risk of carotid plaques in the general population. In contrast to females, an “S”-shaped relationship was observed in males. To our knowledge, this is the first study to investigate gender differences in the relationship between the TyG index and carotid plaque formation. Additionally, our findings indicate that males have a higher risk of developing carotid plaques compared to females.

To date, studies examining the relationship between the TyG index and carotid plaques have shown conflicting results. A community study found no significant correlation between the TyG index and carotid plaque, but its research subjects were over 65 years old, and the weakening effect of long-term use of lipid-lowering and blood sugar-lowering drugs on the correlation between the TyG index and carotid plaque needs to be considered (18). In contrast, another cross-sectional observational study suggested that the TyG index is significantly positively correlated with unstable carotid plaques, but there is no statistically significant association with stable carotid plaques (33). Subsequently, another cross-sectional study pointed out that the TyG index is linearly associated with both stable and unstable carotid plaques (11). However, a study among the Japanese population showed an inverted L-shaped nonlinear correlation between the TyG index and carotid plaques, but no significant gender differences were found (17). Our study identified a nonlinear relationship in the male population. Furthermore, our study builds on previous findings by identifying a gender interaction effect. This suggests that when using the TyG index to assess carotid plaque risk, gender differences must be considered. Specifically, in men, the relationship between the TyG index and carotid plaques follows an S-shaped curve, whereas in women, it is approximately linear.

It is well known that insulin resistance is associated with chronic inflammation, oxidative stress damages vascular endothelium, and may accelerate the progression of atherosclerosis by causing hyperglycemia, dyslipidemia, and other factors (34, 35). The TyG index is also emerging as a novel alternative marker for systemic inflammation (35). In terms of pathophysiology, higher plasma triglyceride levels are more likely to result in endothelial dysfunction, plaque rupture, and arterial inflammation, all of which can lead to arterial plaques (36). Some cohort studies have pointed out that there is a significant dose-response relationship between the TyG index and the risk of arterial stiffness (37, 38). In addition, the TyG index is associated with an increased risk of stroke recurrence, all-cause mortality, and neurological deterioration in patients with ischemic stroke (39). These studies indirectly support our findings that the TyG index may be related to the formation of carotid atherosclerosis.

Our study complements the existing literature by further supporting these viewpoints. In the generalized additive model, we observed that the correlation between TyG and carotid plaque in males is S-shaped compared to females. We hypothesize that this difference may be attributed to the role of genetic polymorphisms in the development of subclinical carotid atherosclerosis in men and the beneficial effects of endogenous estrogen on insulin sensitivity and lipid metabolism in women. Firstly, a cardiovascular risk study showed that the polymorphism of the interleukin-6 promoter gene is associated with markers of subclinical carotid atherosclerosis in males, but not significantly in females (40). Secondly, the mononucleotide polymorphism of the interleukin-6 receptor may be involved in the pathogenesis of carotid atherosclerosis and plaque vulnerability in males (41). Additionally, research on the prevention of atherosclerosis found that estrogen is negatively correlated with the progression of carotid intima-media thickness because it can increase insulin sensitivity and is involved in changing blood lipid levels, such as increasing high-density lipoprotein and reducing low-density lipoprotein (42, 43). These findings can be used to explain the gender differences in the association between the TyG index and carotid plaque, but more future research is needed to further explore the specific mechanisms of gender in this process.

This study also has some limitations. Firstly, this study is cross-sectional, which only demonstrates an association between the TyG index and carotid plaques and does not establish causality. The distinct characteristics observed in men compared to women also require further fundamental research for clarification. Secondly, due to the observational design of our study, we cannot eliminate the possibility of other unmeasured confounding factors. Additionally, since the TyG index is a calculated value based on glucose and lipid levels, we did not adjust for the use of antidiabetic medications in the regression model, which also limits the generalizability of our conclusions. Our analysis involves participants from a single community, which may limit the applicability of our conclusions to other populations.

## Conclusion

In summary, the relationship between the TyG index and the formation of carotid plaques in populations at high risk of cardiovascular disease is nonlinear and exhibits significant gender differences.

## Data Availability

The raw data supporting the conclusions of this article will be made available by the authors, without undue reservation.
