# Supplementary figures and images for: Sex-specific differences in the association between triglyceride glucose index and carotid plaque in a cardiovascular high-risk population: a cross-sectional study based on a Chinese community-dwelling population
Source: Front Cardiovasc Med. 2024 Oct 15;11:1473171. doi: 10.3389/fcvm.2024.1473171 (PMC11523244; doi:10.3389/fcvm.2024.1473171)

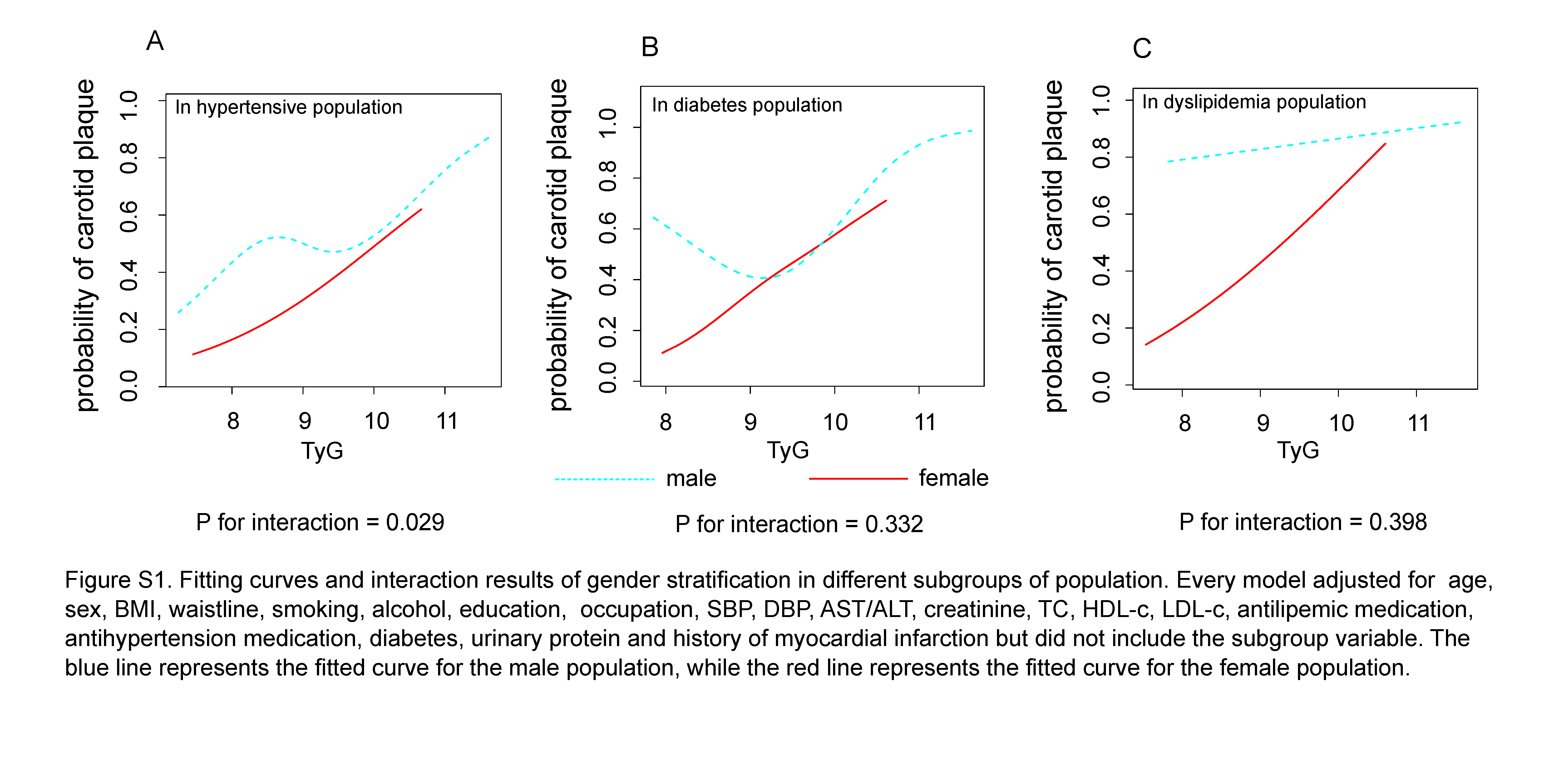

Supplement: Supplementary file 1 [file Image1.tif]
